# Supplementary material for: noisyR: enhancing biological signal in sequencing datasets by characterizing random technical noise
Source: Nucleic Acids Res. 2021 Jun 2;49(14):e83. doi: 10.1093/nar/gkab433 (PMC8373073; doi:10.1093/nar/gkab433)
Supplement: gkab433_Supplemental_Files [file gkab433_supplemental_files.zip › noisyR_submission_supplementary_revision-.docx]

Supplementary Materials for

***noisyR*: Enhancing biological signal in sequencing datasets by characterising random technical noise**

I. Moutsopoulos, L. Maischak, E. Lauzikaite, S. A. Vasquez Urbina, E. C. Williams, H. G. Drost, I. I. Mohorianu*

*Corresponding author. Email: iim22@cam.ac.uk

**This PDF file includes:**

Supplementary methods 1 & 2

Figure captions S1 to S7

Tables S1 and S2

Supplementary method 1

The window length has a significant effect on the signal/noise threshold. For narrow windows, the range of abundances of genes in each window is tight, so small variation (i.e. reshuffling of ranks) causes the correlation to remain low until very high abundances. For wide windows, the correlation becomes a measure of the sequencing depth hindering the identification of signal/noise transition. Empirically, a window length of ~10% of the number of genes provides a good balance; we also developed an approach to infer the window length from the data. First, the distribution of correlations is calculated for varying window lengths (by default between 1% and 33% of the total number of genes). The Jensen-Shannon (JS) divergence is then computed between the distribution of correlations for each window length (subsampled to the same length) and a cumulative uniform distribution. The divergence is high for shorter windows, reaching a plateau as the window length increases. The start of the plateau is then selected as the optimal window length, by comparing the distributions of JS obtained through repeated subsampling. This ensures that stability has been reached, and the selection of the smallest window achieves the optimal accuracy in determining the noise threshold.

**Algorithm 1:** Pseudocode for window length optimisation

**input** the expression count matrix

**parameters** correlation/distance measure for the count matrix approach

window length min, max, and by, used to determine a set of window lengths

step size for the count matrix approach

iteration # for subsampling to calculate the JS

**for** each input *window length* (in decreasing order)

calculate distance matrices using the count matrix approach with that window

calculate the average correlation of each window across samples

**for** each input *iteration* *#*

subsample the mean correlations to a common length
(determined by the largest window length)

calculate the JS divergence between the subsampled vector and the cumulative uniform distribution

**for** each input *window length* (in increasing order)

perform a t-test to determine whether the distribution of JS is similar to that of every other *input window length*

**if** there are two or more similar distributions (stability) **exit**

**output** the window length for which stability was reached

Supplementary method 2

The correlation vs abundance distribution varies across datasets; this phenomenon is accentuated for datasets obtained through different technologies. However, the general, robust conclusion is that low abundances exhibit low correlations, followed by a (rapid) increase in correlation proportional to the abundance increase. We provide options to use a regression of varying smoothness on the data, or create boxplots of local (with regards to abundance) distributions and use those to estimate the noise threshold. With the regression option, the line plot on abundance-correlation is smoothed using the loess function from the stats package in R; the smoothed line is used to determine the abundance threshold as the last time the line exceeds the correlation threshold. For the boxplots, a range for the signal/noise is defined as the last distribution for which the median is lower than the correlation threshold. The correlation threshold, the localization of the distribution and the binning range are user-defined parameters. The localization parameter can be chosen as the median, the lower/upper quartile (25/75%) or top 5%, 95%. We do not recommend the usage of local extremes (min/max) due to the high inter-sample variability. We also recommend a log-base binning approach that will assign at least 25 transcripts per bin for the bins around the mod of the abundance distribution. A correlation threshold is selected and a method that is either a smoothed line plot or a quantile of the boxplots above which the distribution must consistently be is used to determine the corresponding abundance. We recommend using the method that corresponds to the lowest coefficient of variation for the noise thresholds determined across samples, to increase robustness. The boxplots usually perform better where there are many observations, as is the case with the transcript approach, where there is a value for every gene. Using the 25% quantile of the boxplots has shown the best results so we use that by default in the R package. Other approaches to determine the threshold were also considered, such as taking the last negative of the derivative or optimising splines fit to the data, but because the distributions might differ significantly, we would not advise using such methods without good justification.

**Algorithm 2:** Pseudocode for determining the signal to noise threshold

**input** *expression matrix, abundance matrix and correlation/distance matrix*, as output by the count or transcript-based approach

**parameters** *correlation/distance threshold* to use

*method chosen* for the algorithm; if unspecified, all available options are used

*binsize and min. points per box* to be used for the box-plot methods

**if** *method chosen* is density based

**if** *method chosen* includes normalisation

normalise the *expression matrix* using the specified normalisation

log-transform the *expression matrix*

**for** each column of the *expression matrix*

find the first local minimum of the density distribution

calculate the median threshold across all samples

**if** *method chosen* is based on the line plot

**for** each column of the *expression matrix*

**if** *method chosen* includes smoothing

log-transform the *abundance matrix*

perform the smoothing of the *correlation/distance matrix* over the transformed *abundance matrix (corr/dist ~ log(abundance))*

identify the highest abundance for which the correlation/distance (smoothed or not) is below the correlation/distance threshold

**if** *method chosen* is based on the box plot

**for** each column of the *expression matrix*

log-transform the *abundance matrix*

group the abundances into bins using the *binsize* supplied

if any bin has fewer observations than *min. points per box*, group it with the one on its left (by abundance)

using those groupings, calculate the quantile of the *correlation/distance matrix* determined by the *method chosen* (median, etc)

identify the group of the highest abundance for which the calculated quantile is below the correlation/distance threshold

**output** the vector of thresholds by sample determined using the *method chosen*, or (if *method* chosen was unspecified) a summary table of the thresholds by sample and some statistics on them using all available methods.

**Fig. S1 Further review of QC measures and side-by-side assessment of original vs denoised outputs on standard components of an mRNA-seq pipeline.**

**(A)** MA plots of the raw abundances for all pairwise combinations of samples: 0h replicates (top) followed by the four 0h vs 12h comparisons; a larger proportion of low abundance genes exhibit high fold-changes, potentially biasing the DE calls. **(B)** MA plots of the de-noised abundances for all pairwise combinations of samples: 0h replicates (top) followed by the four 0h vs 12h comparisons; the low-level variation is significantly reduced. **(C)** Line plot of the PCC calculated on windows of increasing average abundance for the count matrix-based noise removal approach, for all samples not presented in Fig. 1 **(D)** Volcano plot of differentially expressed genes on the original, normalised count matrix using DESeq2; the colour gradient is proportional to the gene abundance. **(E)** Volcano plot of differentially expressed genes on the denoised count matrix, using DESeq2. **(F)** Histogram of the differentially expressed genes called using DESeq and edgeR respectively, on the original and denoised count matrix, binned by abundance; the z-scores of the differences between original and de-noised output are displayed on the x-axis; the convergence of the two methods is demonstrated by the significant decrease of the specific differences.

**Fig. S2 Results of the noise identification on plant and animal sRNA data.**

The distribution of transcript-based, point-to-point PCC, across miRNA hairpins and transposable elements, is summarised using boxplots on abundance bins. The subplots correspond to plant and animal derived data (represented on the rows, top and bottom, respectively) and hairpins or transposable elements (represented on the columns, LHS and RHS respectively).

**Fig. S3 Further analysis of the impact of noise filtering on smartSeq data.**

**(A)** PCC calculated on windows of increasing average abundance for the count matrix-based noise removal approach applied to the “pseudo-samples” formed by grouping cells randomly. **(B)** UMAP representation of the cells using the raw count matrix grouped by the inferred clusters of the de-noised matrix. **(C)** UMAP representation of the cells using the denoised count matrix grouped by the inferred clusters of the raw matrix. **(D)** Heatmap of the Jaccard similarity index between the 10 most significant markers identified for each cluster on the raw matrix (rows) and denoised matrix (columns). **(E)** Heatmap of the Jaccard similarity index between the 100 most significant markers identified for each cluster on the raw matrix (rows) and denoised matrix (columns).**Fig. S4 Pairwise Hamming distance and edge weight distributions between combinations of original, noise-filtered, and normalised bulk RNAseq datasets.**

Analogous to Fig. 3 F-H, we show that pairwise hamming distance comparisons for each gene between all combinations of original (-F(iltered) -N(ormalised)), noise-filtered (+F), and normalised (+N) input datasets from the bulk RNAseq (Yang et al.) dataset (Methods) show a comparable pattern across different biological pathways with various gene set sizes **(A)** Placenta development, 49 genes; **(B)** Cell differentiation, 249 genes; **(C)** Phosphorus metabolic process, 493 genes; **(D)** Multicellular organism development, 996 genes. Hamming distance computations were performed on rescaled (interval [0,100]) and binarised (median-threshold) adjacency matrices (Methods) and visualised as violin plots with each dot representing the Hamming distance of one gene. All meaningful combinations were performed between input datasets original -F -N; not noise-filtered but normalised -F +N; noise-filtered but not normalised +F -N; and noise-filtered and normalised +F +N. A Kruskal-Wallis Rank Sum Test was performed for comparisons per pathway with resulting p-values (p-value < 0.01, Placenta development; p-value < 0.01, Neuron differentiation; p-value < 0.01, Cell differentiation; p-value < 0.01, Phosphorus metabolic process; p-value < 0.01, Multicellular organism development). **(E-F)** For 102 genes with biological functions associated to neuron differentiation from the bulk RNAseq dataset (Yang et al.), the rescaled (interval [0,100]) network edge weight distributions per gene are shown in two variants: **(E)** without noise-filtering and normalisation (Original) and **(F)** with noise-filtering and normalisation applied (Filtered, Normalised) to illustrate the full spectrum of network interconnectivity before and after noise-filtering/normalisation. The respective networks were inferred using GENIE3 (Methods). The red vertical line denotes the median value derived from all edge-weight values in the weighted adjacency matrix which is subsequently used as threshold to transform this weighted adjacency matrix into a binary adjacency matrix (values below the median threshold are set to zero and weights above the median threshold are set to one).

**Figure S5 Comparison of noisyR signal/noise threshold identification to an exhaustive, incremental search using a fixed threshold approach.**

**(A)** Comparison of consistency of DE genes identification, using the two standard methods - DESeq2 and edgeR, when incremental noise thresholds are used for denoising. We observe a proportional decrease of the total number of DE genes relative to the increased fixed threshold; the reduction mostly affects the specific differences and not genes called DE by both methods. We also highlighted the results obtained using the noisyR threshold which corresponds to a local minimum of the specific differences (sum of green and blue bars).

**(B)** Results of enrichment analyses (on the GO terms, pathways and regulatory elements) performed on the DE genes identified by both methods. We observe a clear maximum that corresponds to the noisyR threshold, which may be the consequence of the larger intersection of DE genes stemming from the usage of sample specific thresholds. These specific thresholds are applied before dataset wide filtering, and perform better in comparison to the fixed threshold applied to all samples.

**Fig. S6 Pairwise Hamming distance and edge weight distributions between combinations of original, noise-filtered, and normalised single-cell RNAseq datasets.**

Analogous to Fig. 7 A-C, we show that pairwise hamming distance comparisons for each gene between all combinations of original (-F(iltered) -N(ormalised)), noise-filtered (+F), and normalised (+N) input datasets from the single-cell RNAseq (Cuomo et al.) dataset (Methods) show a comparable pattern across different biological pathways with various gene set sizes **(A)** Metabolism, 57 genes and **(B)** Cellular Metabolism, 246 genes. Hamming distance computations were performed on rescaled (interval [0,100]) and binarised (median-threshold) adjacency matrices (Methods) and visualised as violin plots with each dot representing the Hamming distance of one gene. All meaningful combinations were performed between input datasets original -F -N; not noise-filtered but normalised -F +N; noise-filtered but not normalised +F -N; and noise-filtered and normalised +F +N. A Kruskal-Wallis Rank Sum Test was performed for comparisons per pathway with resulting p-values (p-value < 0.01, Metabolism; p-value < 0.01, Cellular Metabolism). **(C-D)** For 133 genes corresponding to catalytic activity pathways sampled from the single-cell RNAseq dataset (Cuomo et al.), the rescaled (interval [0,100]) network edge weight distributions per gene are shown in two variants **(C)** without noise-filtering and normalisation (Original) and **(D)** with noise-filtering and normalisation applied (Filtered, Normalised) to illustrate the full spectrum of network interconnectivity before and after noise-filtering/normalisation. The respective networks were inferred using GENIE3 (Methods). The red vertical line denotes the median value derived from all edge-weight values in the weighted adjacency matrix which is subsequently used as threshold to transform this weighted adjacency matrix into a binary adjacency matrix (values below the median threshold are set to zero and weights above the median threshold are set to one).

**Fig. S7 Further impact of aligner choice on noise quantification**

**(A)** The distribution of PCC across abundance bins in datasets for three mRNAseq samples (arranged in the panel from left to right in the following order: 0h rep2, 12h rep1, 12h rep2) obtained by STAR, Bowtie2 and HISAT2 alignment followed by featureCounts quantification using counts-based noise removal approach. **(B)** The distribution of PCC across abundance bins in aligned reads counts obtained by STAR, Bowtie2 and HISAT2 alignment in three mRNAseq samples (arranged in the panel from left to right in the following order: 0h rep2, 12h rep1, 12h rep2) in transcript-based noise correction approach. **(C)** The number of the differentially expressed called by DESeq and edgeR respectively, on the original and denoised (using transcripts-based approach) count matrices obtained by Bowtie2 alignment with default parameters (left plot) and HISAT2 alignment with default parameters (right plot).

Table S1

| Dataset | Term ID | Term name | Number of genes |
| --- | --- | --- | --- |
| Bulk | GO:0001890 | placenta development | 49 |
| Bulk | GO:0045666 | positive regulation of neuron differentiation | 102 |
| Bulk | GO:0045597 | positive regulation of cell differentiation | 249 |
| Bulk | GO:0006793 | phosphorus metabolic process | 493 |
| Bulk | GO:0007275 | multicellular organism development | 996 |
| Single-cell | REAC:R-HSA-1430728 | metabolism | 57 |
| Single-cell | GO:0003824 | catalytic activity | 133 |
| Single-cell | GO:0044237 | cellular metabolic process | 246 |

**Table S1**

Pathways selected to subset genes for GRN inference, on the bulk and single-cell data respectively.

**Table S2**

**(A)**

| # of samples | Count matrix | | Transcript, 10,000 exons | | | |
| --- | --- | --- | --- | --- | --- | --- |
|  | runtime (min) | genes retained | runtime, ncores=1 (min) | runtime, ncores=16 (min) | runtime, ncores=32 (min) | genes retained |
| 2 | 0.01 | 24,231 | 26.77 | 1.36 | 1.30 | 19,961 |
| 4 | 0.03 | 25,578 | 58.69 | 2.27 | 2.19 | 20,221 |
| 6 | 0.05 | 24,611 | 99.65 | 3.09 | 3.17 | 20,082 |
| 8 | 0.10 | 25,027 | 146.77 | 4.29 | 4.09 | 20,947 |
| 10 | 0.14 | 22,634 | 205.69 | 5.10 | 5.03 | 22,022 |
| 12 | 0.20 | 21,616 | 249.01 | 6.80 | 5.92 | 21,988 |
| 14 | 0.26 | 20,414 | 297.81 | 12.05 | 6.90 | 22,510 |
| 16 | 0.33 | 19,664 | 348.24 | 13.95 | 7.83 | 22,680 |

**(B)**

| # of exons | Count matrix | | Transcript | | | |
| --- | --- | --- | --- | --- | --- | --- |
|  | runtime (min) | genes retained | runtime, ncores=1 (min) | runtime, ncores=16 (min) | runtime, ncores=32 (min) | genes retained |
| 100 |  |  | 1.29 | 0.10 | 0.14 | 26,630 |
| 500 |  |  | 15.17 | 0.53 | 0.49 | 20,782 |
| 1,000 |  |  | 25.25 | 0.86 | 0.75 | 21,193 |
| 5,000 |  |  | 130.71 | 4.17 | 3.66 | 21,602 |
| 10,000 |  |  | 283.26 | 9.29 | 7.89 | 22,680 |
| 50,000 |  |  |  | 51.72 | 37.52 | 23,504 |
| 100,000 |  |  |  | 99.53 | 75.45 | 23,706 |
| 449,800 (all) |  |  |  | 513.86 | 398.55 | 23,568 |

**(C)**

| # of uniquely mapped reads per sample | Count matrix | | Transcript, 10,000 exons | | | |
| --- | --- | --- | --- | --- | --- | --- |
|  | runtime (min) | genes retained | runtime, ncores=1 (min) | runtime, ncores=16 (min) | runtime, ncores=32 (min) | genes retained |
| 5M |  |  | 68.34 | 6.83 | 3.83 | 26,035 |
| 15M |  |  | 140.15 | 8.62 | 4.84 | 25,810 |
| 30M |  |  | 227.62 | 10.97 | 6.16 | 23,814 |
| 37-57M (all) |  |  | 290.45 | 14.00 | 7.86 | 22,680 |

**Table S2** **Benchmarking results of noisyR runtime**.

We performed computational benchmarks for both the count-based and the transcript-based approaches, with several covariates being assessed, using the Yang et a 2019 dataset. For the transcript approach, we varied also the number of cores used for parallel processing n = 1, 16, 32. Each table contains the same reference case study, highlighted in blue. **(A)** Effect of varying the number of samples from two to 16; the transcript approach was limited to 10,000 exons to avoid excessive runtimes. The count matrix approach is orders of magnitude faster across all case studies; increasing the number of cores for the transcript approach significantly, proportionally reduces the runtime. We observe, across approaches, comparable numbers of retained genes, but opposite trends. For the count-matrix approach, the relative order of low abundance genes can vary in each sample, so adding more samples reduces the overall similarity. For the transcript approach, each gene is assessed individually, i.e. adding more samples increases the consistency of expression patterns for genes close to the noise threshold, thus excluding fewer of them. **(B)** Effect of varying the number of exons, only applicable on the transcript-based approach; we illustrate the runtimes for 100 to 449,800 exons (i.e. all exons present in the gtf file for the *M. Musculus* genome - GRCm38 / mm10). The benchmarking with one core was stopped at 10,000 exons due to excessive runtimes. We observe a proportional increase in runtime with the number of exons; the usage of multiple cores mitigates this increase. We also observe a convergence of the number of genes retained as we increase the number of exons considered. **(C)** Effect of varying the sequencing depth/number of mapped reads, only applicable on the transcript-based approach. The subsampling was performed on the BAM files (on uniquely mapped reads, the explored range was 5M reads to the original sequencing depth, 37M - 57M across samples). The benchmarking was done with 10,000 exons to avoid excessive runtimes. We observe a non-linear reduced runtime for lower sequencing depth, as each gene is still processed even when it is expressed at low levels or not at all. We also note an increase of the number of retained genes for lower sequencing depth, due to the high proportion of zero values across the transcripts; highly expressed genes are largely unaffected.
