# Supplementary figures and images for: noisyR: enhancing biological signal in sequencing datasets by characterizing random technical noise
Source: Nucleic Acids Res. 2021 Jun 2;49(14):e83. doi: 10.1093/nar/gkab433 (PMC8373073; doi:10.1093/nar/gkab433)

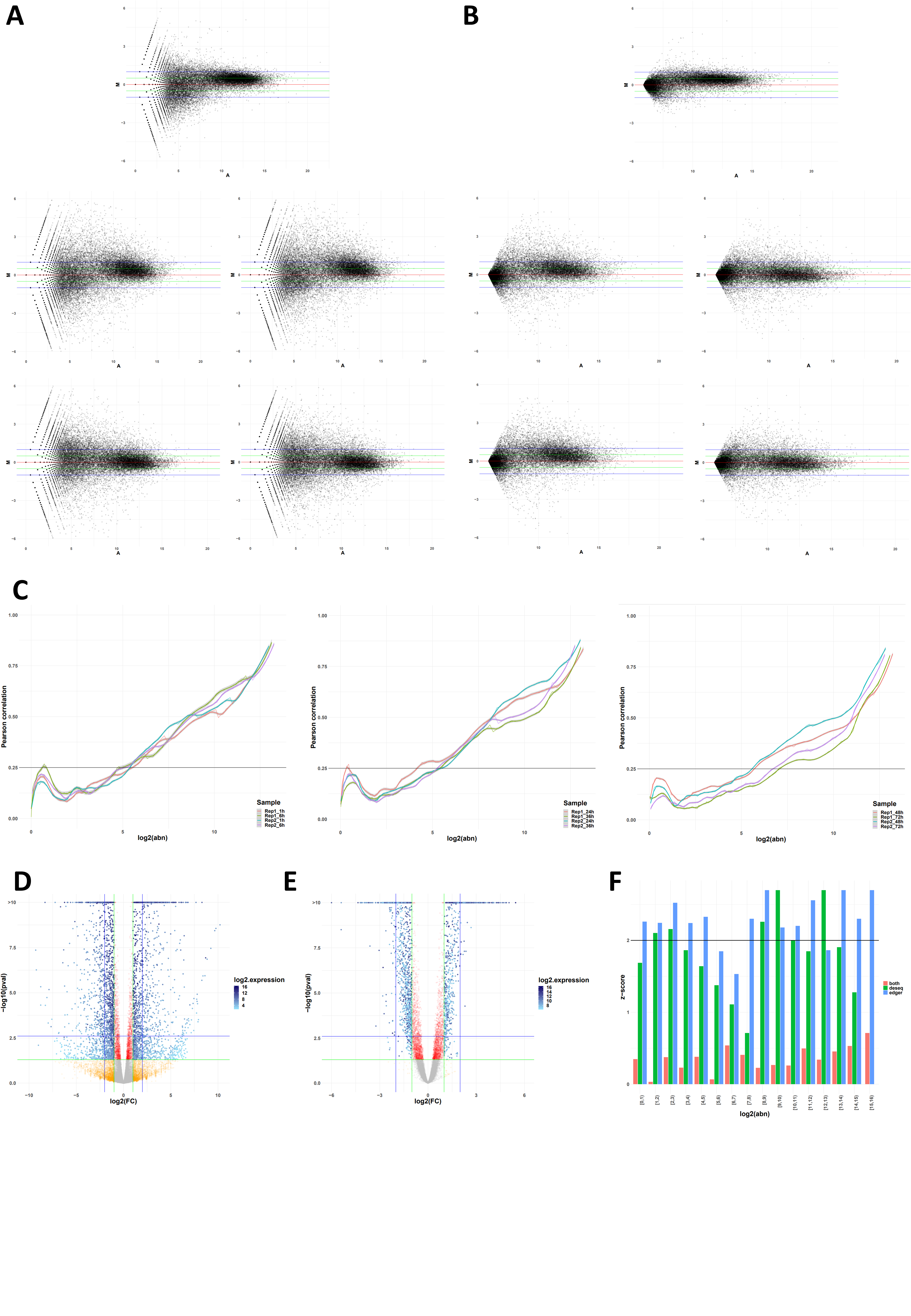

Supplement: gkab433_Supplemental_Files [file gkab433_supplemental_files.zip › FigS1.tif]

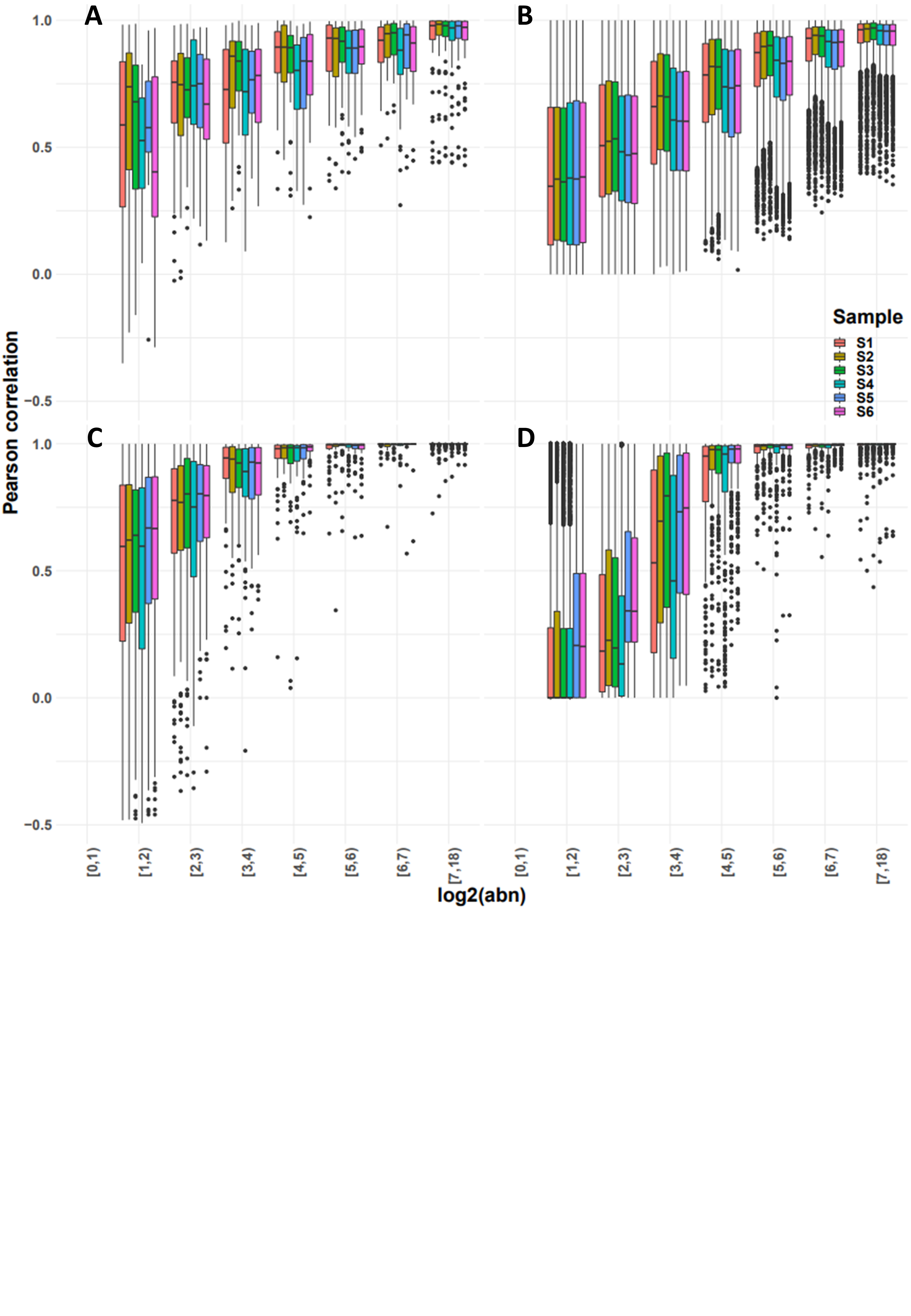

Supplement: gkab433_Supplemental_Files [file gkab433_supplemental_files.zip › FigS2.tif]

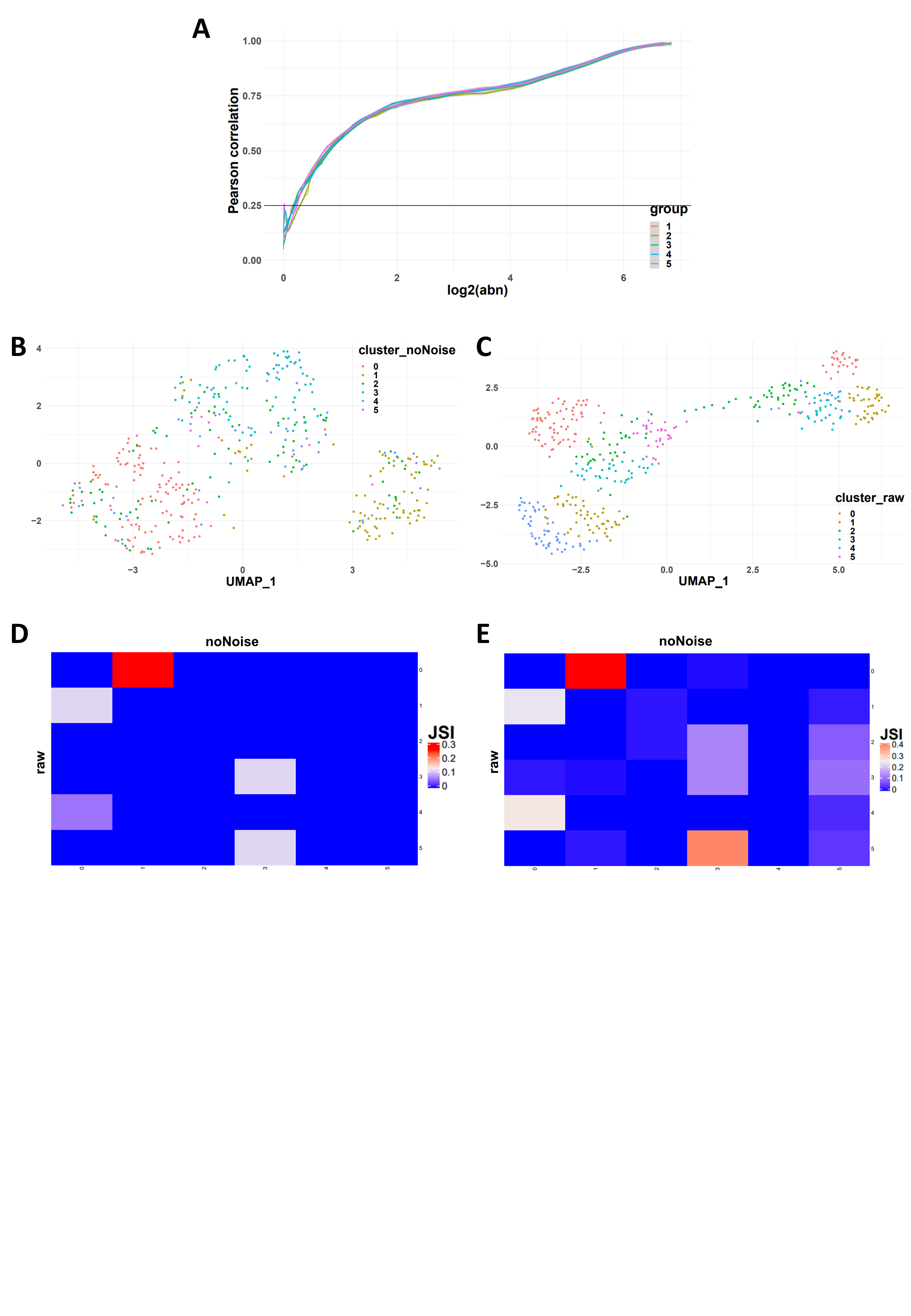

Supplement: gkab433_Supplemental_Files [file gkab433_supplemental_files.zip › FigS3.tif]

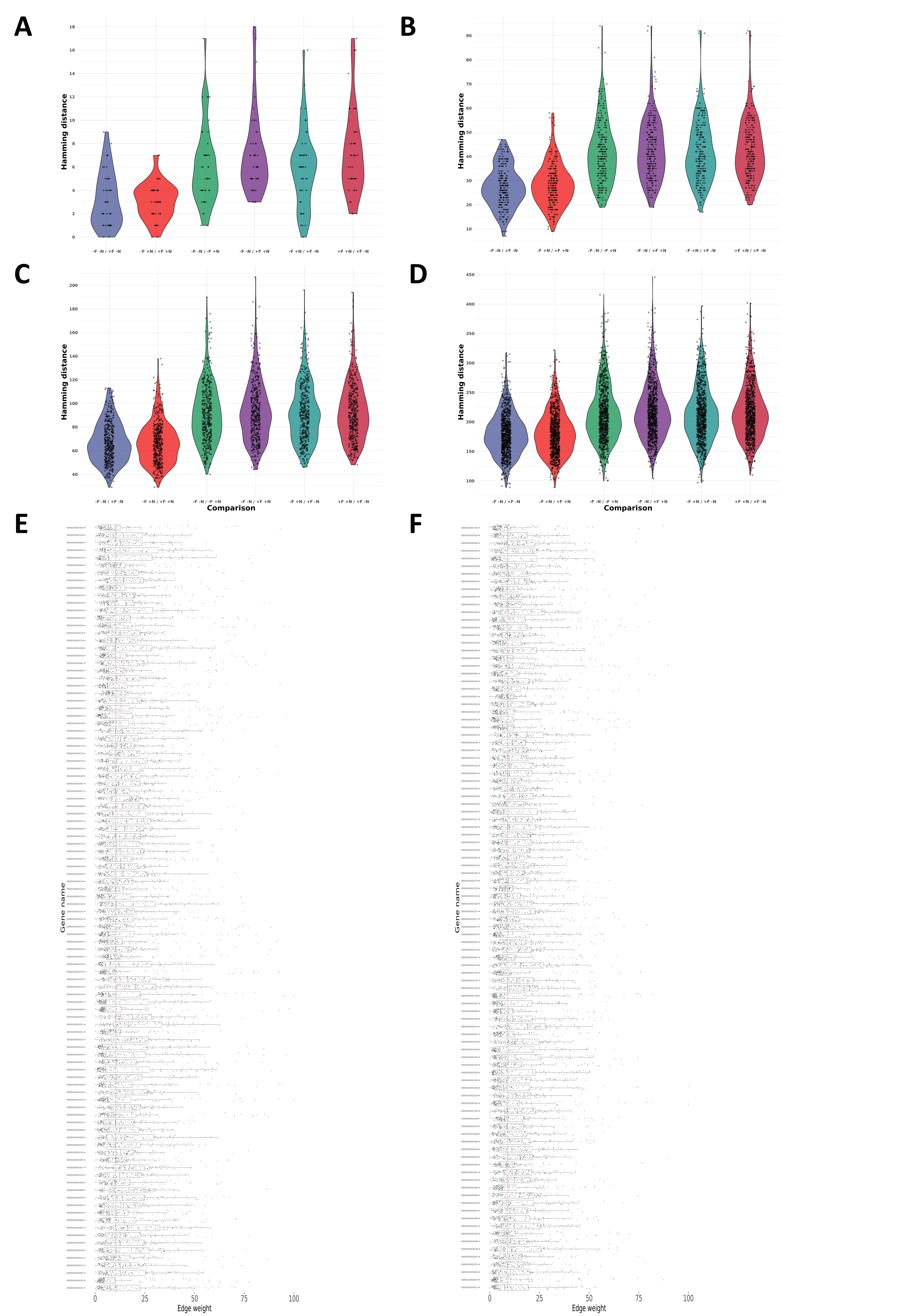

Supplement: gkab433_Supplemental_Files [file gkab433_supplemental_files.zip › FigS4.tif]

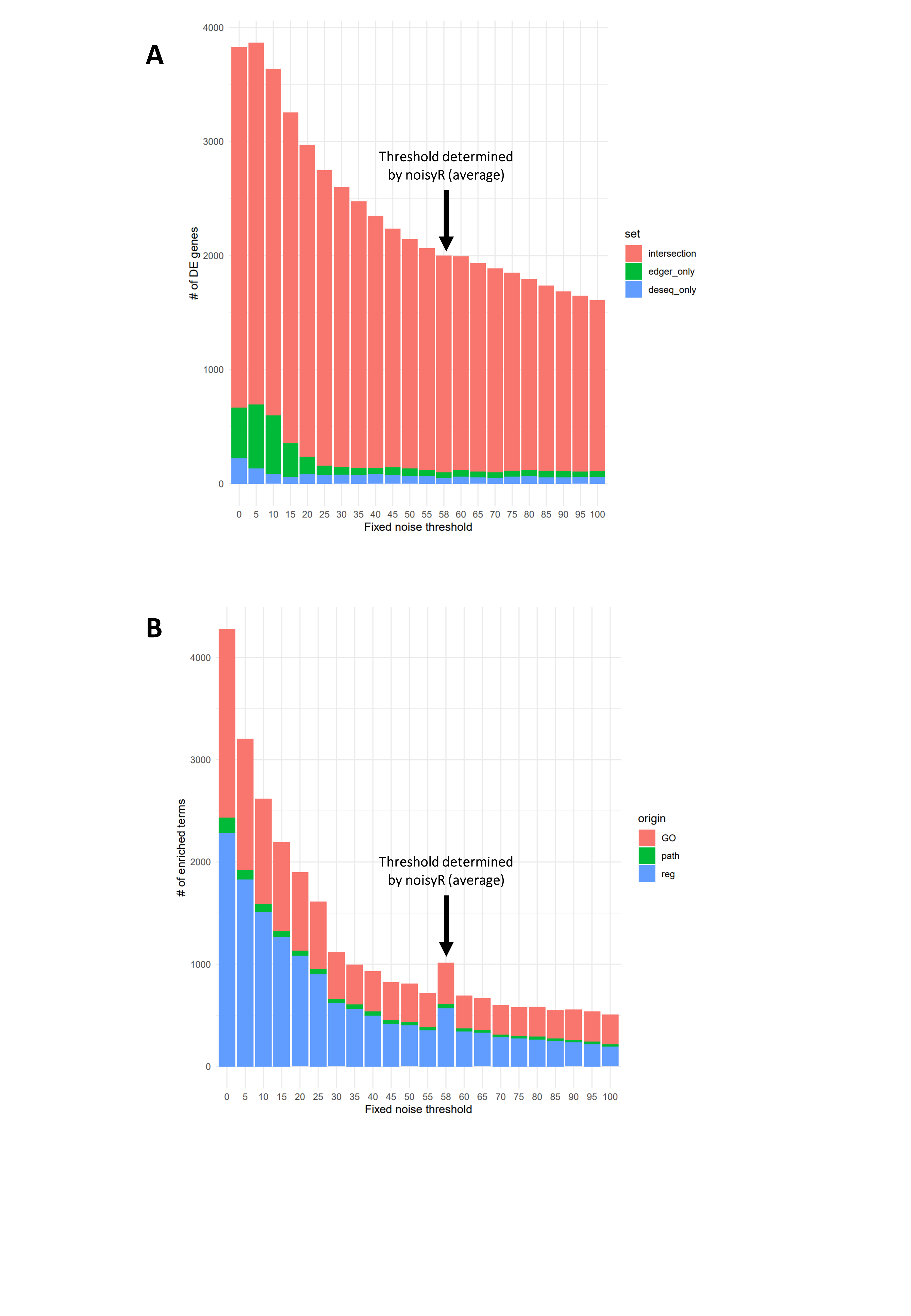

Supplement: gkab433_Supplemental_Files [file gkab433_supplemental_files.zip › FigS5.tif]

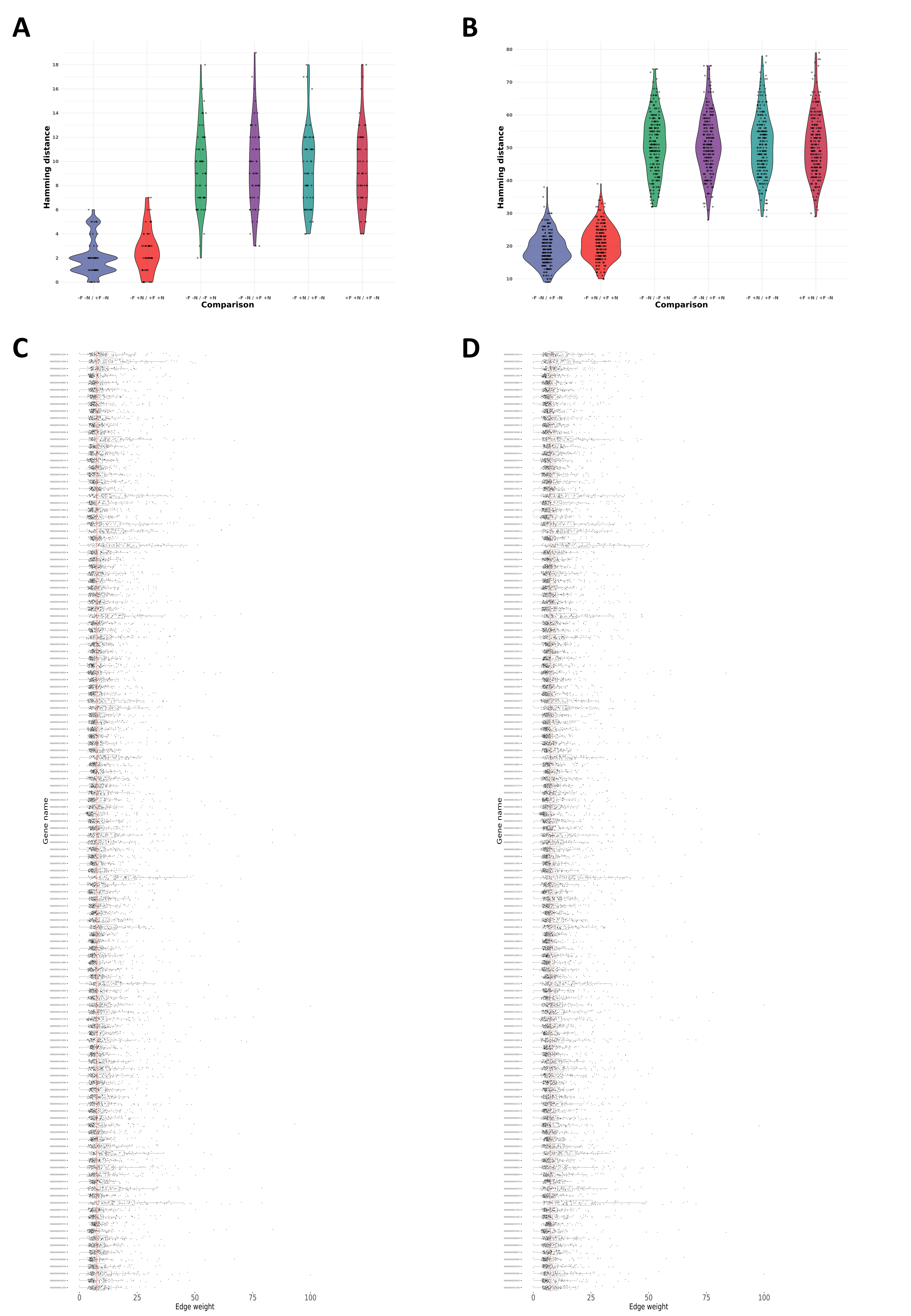

Supplement: gkab433_Supplemental_Files [file gkab433_supplemental_files.zip › FigS6.tif]

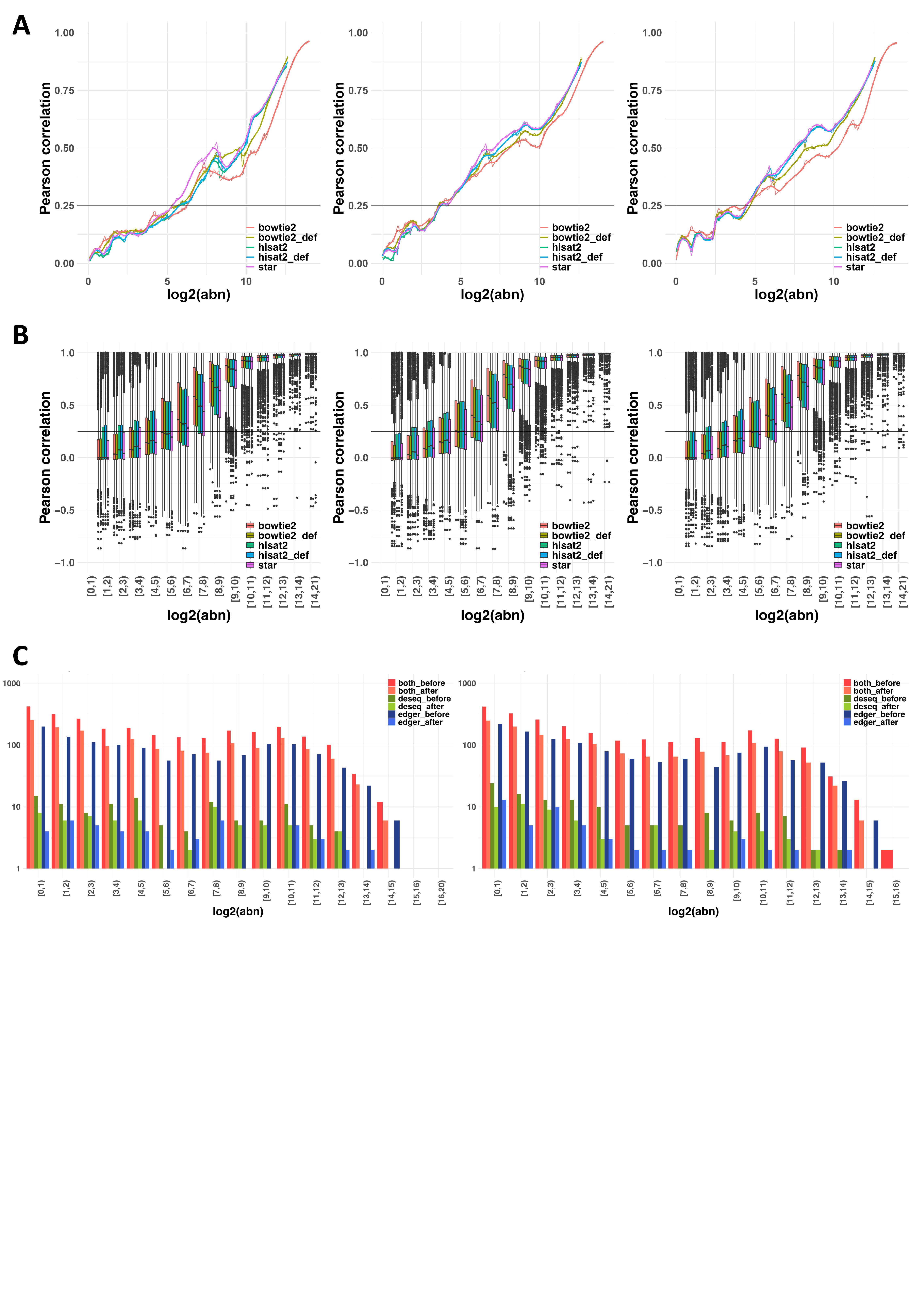

Supplement: gkab433_Supplemental_Files [file gkab433_supplemental_files.zip › FigS7.tif]
